# Genome assembly of *Nannochloropsis oceanica* provides evidence of host nucleus overthrow by the symbiont nucleus during speciation

**DOI:** 10.1038/s42003-019-0500-9

**Published:** 2019-07-03

**Authors:** Li Guo, Sijie Liang, Zhongyi Zhang, Hang Liu, Songwen Wang, Kehou Pan, Jian Xu, Xue Ren, Surui Pei, Guanpin Yang

**Affiliations:** 1 College of Marine Life Sciences, Ocean University of China (OUC), Qingdao, 266003 P. R. China; 2 College of Agriculture and Resources and Environment, Tianjin Agricultural University, Tianjin, 300384 P. R. China; 3 Laboratory of Applied Microalgae, College of Fisheries, OUC, Qingdao, 266003 P. R. China; 4 Functional Genomics Group, Qingdao Institute of Bioenergy and Bioprocess Technology, Chinese Academy of Sciences, Qingdao, 266101 P. R. China; 5 Annoroad Gene Technology (Beijing) Co., Ltd, Beijing, 100176 P. R. China; 6 Institutes of Evolution and Marine Biodiversity, OUC, Qingdao, 266003 P. R. China; 7 Key Laboratory of Marine Genetics and Breeding of Ministry of Education, OUC, Qingdao, 266003 P. R. China

**Keywords:** Genomics, Evolution

## Abstract

The species of the genus *Nannochloropsis* are unique in their maintenance of a nucleus-plastid continuum throughout their cell cycle, non-motility and asexual reproduction. These characteristics should have been endorsed in their gene assemblages (genomes). Here we show that *N. oceanica* has a genome of 29.3 Mb consisting of 32 pseudochromosomes and containing 7,330 protein-coding genes; and the host nucleus may have been overthrown by an ancient red alga symbiont nucleus during speciation through secondary endosymbiosis. In addition, *N. oceanica* has lost its flagella and abilities to undergo meiosis and sexual reproduction, and adopted a genome reduction strategy during speciation. We propose that *N. oceanica* emerged through the active fusion of a host protist and a photosynthesizing ancient red alga and the symbiont nucleus became dominant over the host nucleus while the chloroplast was wrapped by two layers of endoplasmic reticulum. Our findings evidenced an alternative speciation pathway of eukaryotes.

## Abstract (web summary)

Li Guo et al. assemble the genome of *Nannochloropsis oceanica*, finding evidence that during the secondary endosymbiosis event responsible for its speciation, its host nucleus was overthrown by a symbiont nucleus. They suggest that the symbiont nucleus became dominant over the host nucleus.

## Introduction

Species in the genus *Nannochloropsis* are promising for industrial applications because they accumulate large quantities of polyunsaturated fatty acids^1,2^. They also show promising characteristics allowing convenient genetic improvement, which include, for example, a monoploid nucleus and asexual reproduction, as revealed by mutation^3^, homologous recombination^4^, and preliminary genome sequencing^5^. They are easy to genetically manipulate. Various species of the genus *Nannochloropsis* are able to perform homologous recombination^4^ and genome editing^6,7^. The species in genus *Nannochloropsis* are mainly used as feed for fish larvae and rotifers, and as food additives for human nutrition. They have also evolved gradually into the models for both industrial applications and biological research^8,9^.

Genus *Nannochloropsis* comprises six known species that inhabit marine, freshwater and brackish environments^10,11^. Recently, a phylogenetic analysis of the concatenated 18S ribosomal RNA (rDNA) and rbcL for *Nannochloropsis* taxon revealed a new species, *N*. *australis*, and erected a new genus, *Microchloropsis*, comprising *M*. *salina* and *M*. *gaditana*, which were modified from *N*. *salina* and *N*. *gaditana*, respectively^12^. Whether they belong to *Nannochloropsis* or *Microchloropsis*, the species in these taxa are assigned to class Eustigmatophyceae, phylum Ochrophyta, superphylum Heterokonta (or Stramenopiles)^10^. Heterokonta refers to flagellated cells with two differently shaped flagella. Of the species in Heterokonta, the colored alga-like species in phylum Ochrophyta include diatoms and golden, brown, and yellow-green algae, among others. However, all of the species in the genera *Nannochloropsis* and *Microchloropsis* are small, nonmotile, and spherical in shape^13^; they can be distinguished only by their rbcL and rDNA sequences^14–16^. The algae of genus *Nannochloropsis* are able to build up high concentrations of diverse pigments, such as astaxanthin, zeaxanthin and canthaxanthin^17^ and have chlorophyll *a* but completely lack chlorophylls *b* and *c*^18^.

The genomes of two species in the genus *Nannochloropsis*, *N. gaditana*^19,20^ and *N. oceanica*^21,22^, have been sequenced on the Illumina platform. Their genomes vary between 28.5 and 29.0 Mb in size and are characterized by a high density of genes, low intron content, short spacers between genes^19^ and few repetitive sequences^21^. In addition, a comparison of the genomes of all six species in the genus *Nannochloropsis* revealed an extreme case of dose expansion among lipid biosynthesis genes^23^. These genomes are all sequenced on Illumina platforms, and have not been scaled up to pseudochromosomes with the newly commercialized technologies, including single-molecule real-time (SMRT) sequencing^24^ and Hi-C technology^25,26^, among others. Such scenario is detrimental to identify completely the genes each genome.

We noted in particular that the ultrastructure of *N. oculata* is unusual; a nucleus-plastid continuum exists throughout its cell cycle^27^, but the basal body and flagellum observable in green algae^28^ are absent. Considering comprehensively the above findings, we suspected that an invaded red algal nucleus may have overthrown the host protist nucleus during evolution, i.e., a red algal nucleus has occupied the host cytoplasm, and the host nucleus has been expelled. Unfortunately, these unique characteristics have not been deciphered genetically and evolutionarily.

In this study, we sequenced the genome of *N. oceanica* with a real-time DNA sequencing method and scaled up the primary assembly with the chromatin conformation information revealed by Hi-C data, aiming to look for evidence supporting our assumptions. A genome assembly of 29.3 Mb containing 32 chromosome-scale scaffolds (pseudochromosomes) was obtained. The genome contains 7330 protein-coding genes, and most of them may originate from an ancient red alga symbiont nucleus through secondary endosymbiosis during speciation. We also found that *N. oceanica* has adopted a genome reduction strategy during speciation; it has lost its flagella and abilities to undergo meiosis and sexual reproduction. We propose that *N. oceanica* emerged through the active fusion of a host protist and a photosynthesizing ancient red alga and the symbiont nucleus became dominant over the host nucleus, while the chloroplast was wrapped by two layers of endoplasmic reticulum. Our findings evidenced an alternative speciation pathway of eukaryotes.

## Results

### Genome assembly and characterization

**Fig. 1:**
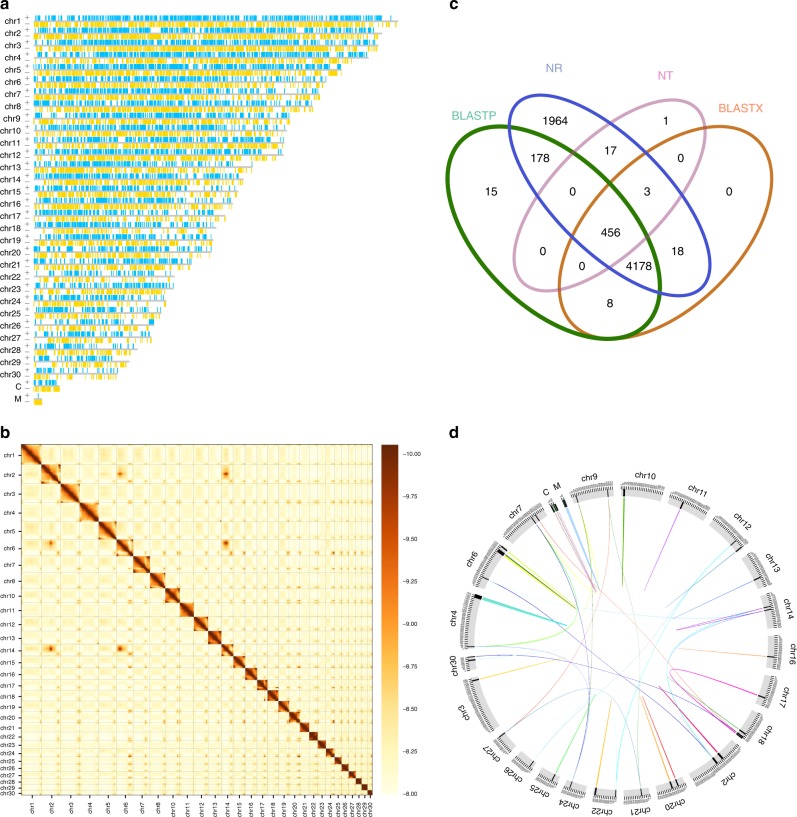
Protein-encoding gene models on each pseudochromosomes of *N. oceanica*. Mitochondrial and chloroplast pseudochromosomes are presented linearly but they can be circulated as their two ends contain the repeat assemblies of sequences. **a** The pseudochromosomes with gene models marked with vertical bars. The green bars represent gene models in the sense strand, while the yellow ones in the anti-sense strand of DNA. **b** The heatmap showing the interaction between 30 nuclear pseudochromosomes. **c** The Venn diagram of functional genes annotated against NT, NR, BLASTX, and BLASTP databases. **d** The similarity between nuclear pseudochromosomes. Less homozygosity is found among these pseudochromosomes, which is one of the characteristics of *N. oceanica*, genome reduction

We sequenced the genome of *N. oceanica* with a third-generation sequencing technique. The quality reads were assembled into contigs that were then grouped, ordered and oriented into pseudochromosomes based on Hi-C revealed chromatin conformational information. In total, 3.31 G raw PacBio reads (4.3 kb in average length, ~113-fold of the genome) were generated on the Sequel System, and 773,137 clean reads were obtained. The PacBio reads were self-corrected and assembled with Canu and polished using PacBio reads and Illumina reads (~105-fold of genome), and then assembled into 129 contigs with a contig N_50_ of 664.75 kb. The longest contig was 1.54 Mb in length. The assembled genome was 29.3 Mb in total length. The GC content of the genome was 54.01%. To evaluate the integrity of the assembly, the assembly was assessed with 303 Benchmarking Universal Single Copy Orthologs (BUSCO)^29,30^ genes from eukaryota ortholog database, of which 264 (87.1%) were annotated and 255 (84.2%) were intact. In order to anchor the 129 contigs into pseudochromosomes, the Hi-C library was constructed and ~425.8 million reads were generated on Illumina platform. In total, ~201.7 million valid interaction reads were used to build the interaction matrices and draw the heatmap. Eighty six contigs were ordered as trunks which contained 26,395,858 bp (~93.84%) and the others were reinserted in between the trunks to maximize the amount of linkage. As results, 128 contigs were ordered (28,129,022 bp, ~95.99%) and these contigs have high-confidential orientations within each chromosome-scale group as were calculated with weighted directed acyclic graph (WDGA). With the assistance of Hi-C data, the primary genome assembly was scaled up to 32 pseudochromosomes (pseudochromosomal N_50_ = 1.14 Mb), including 30 nuclear, one mitochondrial, and one chloroplast pseudochromosomes (Table 1, Supplementary Tables 1–4, Fig. 1a–c). The possible errors revealed by the heatmap showing interactions among the pseudochromosomes (Fig. 1b) may result from the contigs containing high portions of repeats rather than from the order and orientation of the contigs themselves.

**Table 1:** The characteristics of *N. oceanica* genome

Assembled genome size (bp)	29,303,273
Read coverage depth	112×
No. of contigs	129
Contig N_50_ (bp)	664,749
Length of maximum contig	1,540,838
No. of contigs clustered	129
No. of contigs ordered and oriented	128^a^
No. of contigs in trunks	86^b^
No. of pseudochromosomes	32
No. of nuclear chromosomes	30
No. of chloroplast chromosome	1
No. of mitochondrial chromosome	1
Total length of pseudochromosomes (bp)	29,303,273
Max. length of pseudochromosomes (bp)	1,670,642
No. of protein-coding genes	7330
Average length of protein-coding genes (bp)^c^	2084
Length percentage of repeat sequence (bp)	19.54
Length percentage of non-protein-coding genes (bp)	0.0674
No. of exons each gene	2.87
Average length of exons (bp)	483.7
Average length of introns (bp)	370.9

^a^One contig is a singleton, which is not related with any other contigs

^b^A trunk contains at least more than three contigs

^c^A portion of these genes are annotated as hypothetical

Gene modeling and homolog searching identified 7330 nonredundant protein-coding genes. The exons of these genes were 483.7 bp in average. The introns of these genes were 370.9 bp in average. The number of exons in each gene was 2.87. Different numbers of non-protein-coding miRNA, tRNA, rRNA, and snRNA genes were also predicted, which in total accounted for 0.0674% of the genome. The repeated sequences occupied ~19.52% of the genome (Table 1; Supplementary Tables 5–8).

The genome assembly of *N. oceanica* contained 7330 protein-coding genes, which were 2084 bp in average. Unfortunately, a portion of these genes were annotated as putative/hypothetical protein-coding genes. It was interesting to note that 1030 genes (14.05%) contained 2354 transposons, ~2.26 transposons each. This result was different from those in higher plants where retrotransposons are the greatest contributors to the size expansion of the genome^21^. The transposons in the genome of *N. oceanica* were fewer in number, indicating reduction of *N. oceanica* genome (Table 1; Supplementary Table 9; Fig. 1d). The high-quality genome assembly ensured that searching for homologous genes and phylogenetic analyses of both genomes and genes can be carried out appropriately.

### Phylogenetic position of *N. oceanica* within Heterokonta

**Fig. 2:**
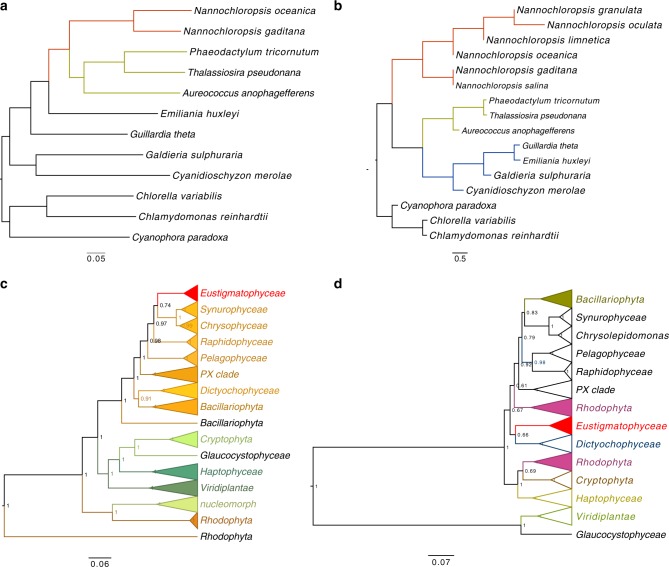
Phylogeny of *Nannochloropsis* and their relatives in different taxa deduced from nuclear genome (**a**), chloroplast genome (**b**), 18S ribosomal RNA gene (18S rDNA, **c**) and ribulose bisphosphate carboxylase large chain gene (*rbcL*) (**d**). **a** The phylogenetic tree of species inferred from the OrthoFinder using the genome protein sequences each species from the whole genome. **b** The phylogenetic tree of species inferred from the OrthoFinder using the chloroplast genome sequences each species. **c** The phylogenetic trees for 18S rDNA. The tree shows the consensus tree topology inferred by Bayesian analysis using alignments of 18S genes from NCBI. The scale bar indicates the nucleotide substitutions per site. This consensus topology derived from 512 trees, ln*L* = 22033.73. **d** The phylogenetic trees for rbcL protein. The tree shows the consensus tree topologies inferred by Bayesian analysis using alignments of rbcL proteins from NCBI. Scale bars represent 0.1 amino acid substitutions per site. In total, 439 aligned amino acid sites were analyzed. This consensus topology derived from 726 trees, *α* = 0.47 (0.41 < *α* < 0.56), *pI* = 0.0019 (0.0000007 < *pI* < 0.0059) and ln*L* = 10364.8

The phylogenetic tree of 18S ribosomal RNA gene (rDNA) sequences representing Rhodophyta, Chromista, Viridiplantae, and Glaucophytes, among others, showed that *Nannochloropsis* were at the top of the current Chromista (Fig. 2c; Supplementary Fig. 1), which were similar to some peridinin-containing dinoflagellates evolved through tertiary endosymbiosis. The nuclei involved in diverse endosymbioses belong to different protists. The positions of *Nannochloropsis* on the phylogenetic tree of 18S rDNA were as expected and demonstrated that the nuclei of *Nannochloropsis* are host ones. It was expected that the vast majority of *Nannochloropsis* protein-coding genes could be placed at the position of rDNA. However, we found that the whole-genome-based microevolution of Heterokonta showed a parallel relationship between *Nannochloropsis* and the microalgae derived through the second endosymbiosis (Fig. 2a), indicating that the vast majority of *Nannochloropsis* protein-coding genes are phylogenetically different from rDNA. This discrepancy implied that *Nannochloropsis* nuclei have different origins from those of other Chromista.

**Fig. 3:**
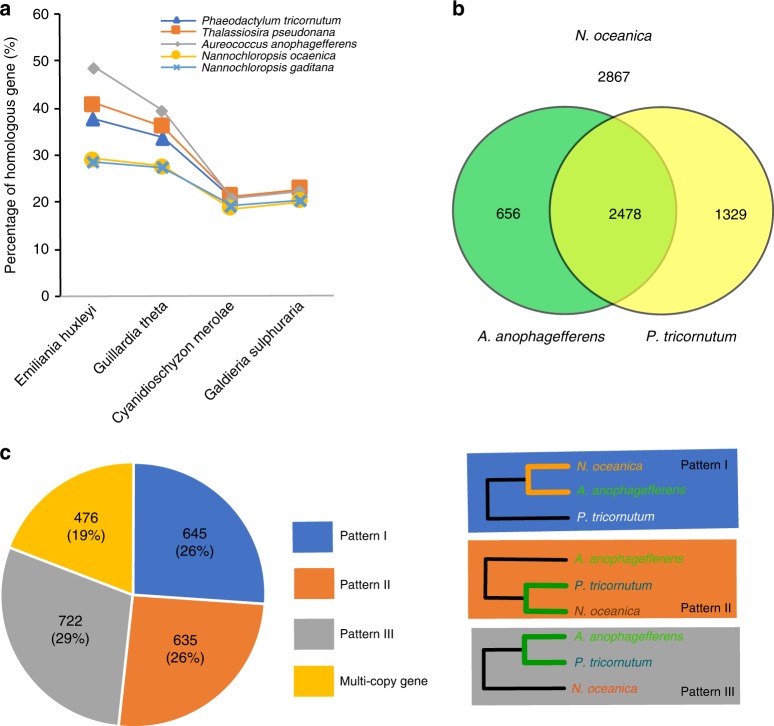
Homologous protein-coding genes of *N. oceanica* found among a few representative microalgal species. **a** The percentages of homologous genes of *P. tricornutum*, *T. pseudonana*, *A. anophagefferens*, *N. oceanica*, and *N. gaditana* found in the nuclear genomes of *E. huxleyi*, *G. theta*, *C. merolae*, and *G. sulphuraria*. **b** The homologous protein-coding genes of *N. oceanica* found in *A. anophagefferens* but not in *P. tricornutum*, in *P. tricornutum* but not in *A. anophagefferens* and in both of them. **c** The number and percentage of the homologous genes of *N. oceanica* found in both *A. anophagefferens* and *P. tricornutum* (2478 in total) can be further partitioned into three patterns (I through III) and multi-copy genes. The pattern I contains the genes phylogenetically near to those of *A. anophagefferens*, while patterns II and III contain the genes phylogenetically far from those of *A. anophagefferens*

We calculated the percentages of homologous genes of two diatoms, one golden microalga and two *Nannochloropsis* in *E. huxleyi*, *G. theta* (nucleus) and two single-cell red algae (Fig. 3a). We found that the percentages of genes homologous to those of red algae are similar among *Nannochloropsis* and other Chromista, while the percentages of genes homologous to those of Haptophytes and Cryptophytes are obviously different (Fig. 3a). The percentages of *Nannochloropsis* genes with homologs in Haptophytes and Cryptophytes are obviously lower than those of diatoms and golden microalgae, indicating that a portion of *Nannochloropsis* genes have been replaced by those from the symbiont nucleus. When we calculated the microevolutionary tree, OrthoFinder simultaneously generated a list showing the homologs among species used to calculate the tree. We selected three species, *N. oceanica*, *A. anophagefferens*, and *P. tricornutum*, as data points; their genome sequences are currently available, and their positions on the 18S rDNA phylogenetic tree are appropriate for determining the sources of genes. We counted the homologs among *N. oceanica*, *A. anophagefferens*, and *P. tricornutum*. Of 7330 protein-coding genes of *N. oceanica*, 2867 genes were unique, and 4463 genes were homologous to *A. anophagefferens* (656) or *P. tricornutum* (1329) only or to both of them (2478) (Fig. 3b). Judging from the number of homologous genes, the nucleus of *N. oceanica* is more similar to that of *P. tricornutum* and, thus, more distant from that of *A. anophagefferens* (1329 vs. 656). Except for the microevolutionary tree, the OrthoFinder calculated also the phylogenetic trees for the homologs using BLAST, MAFFT, and FastTree as the tools and green algae and Glaucophytes as outgroup. A gene of *N. oceanica* is from the host nucleus if it is phylogenetically near its homolog from *A. anophagefferens* (Pattern I; Fig. 3c), while a gene of *N. oceanica* is from the symbiont nucleus if it is phylogenetically far from its homolog in *A. anophagefferens* (Patterns II and III, Fig. 3c). In order to clarify the evolutionary relationships of 2478 *N. oceanica* proteins with homologs in both *A. anophagefferens* and *P. tricornutum*, we aligned the amino acid sequences of these proteins one by one by setting that a cluster must contain only two sequences, and found that 645 (26%) belonged to Pattern I, 635 (26%) to Pattern II, and 722 (29%) to Pattern III. The remaining 476 cannot be categorized into these patterns; they are encoded by multiple copy genes (Fig. 3c). The genes in pattern I were phylogenetically near *A. anophagefferens*, indicating their host nuclear origin, while those in patterns II and III were phylogenetically far from *A. anophagefferens*, indicating their symbiont nuclear origin. Judging again from the number of genes homologous to those of both *A. anophagefferens* and *P. tricornutum*, *Nannochloropsis* nuclei are different from those of *A. anophagefferens* (1357 vs. 645). Therefore, we believe that the *Nannochloropsis* nuclei derive mainly from symbiont nuclei.

The protoplasts of Chromista are believed to have a single ancient origin^31,32^. However, we found that the phylogenetic position of class Eustigmatophyceae is unusual. As indicated by the phylogenetic tree of the rbcL, families Eustigmatophyceae and Dictyochophyceae clustered separately from single-cell red algae and Chromista (Fig. 2d; Supplementary Fig. 2). The whole-genome-based microevolutionary analysis showed that *Nannochloropsis* formed a clade outside the single-cell red algae (Fig. 2b). These findings demonstrated that the symbiont carries an ancient red algal chloroplast. Accordingly, we believe that the symbiont is an ancient red alga that is phylogenetically distant from the current single-cell microalgae.

### Simple protein import mechanism into the plastid

We have extensively searched the genome of *N. oceanica* obtained in this study and those of *N. gaditana*, *P. tricornutum*, *C. merolae*, and *G. sulphuraria*, which were published previously, for the protein components of translocons (or complexes) mediating protein import across the four membranes covering their plastids using the identified proteins^33,34^ as the queries. We found that the major components of SELMA complexes were absent in the two species of *Nannochloropsis*, and a few residual proteins had no predictable bipartite topogenic signal except sDer1-1 and sHsp70 of *N. oceanica* (Table 2). We also found that TOC75, the main component of the import complex across the outer membrane of plastids, was absent in *N. oceanica*, while its alternative, Omp85, existed (Table 3). In addition, the main component of the import complex across the inner membrane of plastids, Tic110, was found to exist in *N. oceanica* but not in *N. gaditana*, which may be due to the incompleteness of its genome sequence. All these findings indicated that *N. oceanica* and *N. gaditana* have either evolved a different protein import system from that of *P. tricornutum* or do not need these complexes at all.

**Table 2:** The identification of host ERAD (endoplasmic reticulum-associated protein degradation) and symbiont SELMA (symbiont-derived ERAD-like machinery) components in *N. oceanica* (NO), *N. gaditana* (NG), *P. tricornutum* (PT), *C. merolae* (CM), and *G. sulphuraria* (GS)

Species		NO^a^		NG^a^		PT^a^		CM^a^	GS^a^
		H	S^?^	H	S^?^	H	S		
ER translocation	Sec61	X	−	X	−	X	−	X	X
Derlin protein	Dfm1, hDer1-1 (sDer1-1)	X	X^sp^	X	X	X	X	X	X
	Der1, hDer1-2 (sDer1-2)	X	−	X	−	X	X	X	X
Ubiquitinylation	Hrd1/Der3 (ptE3p)	X	−	X	−	X	X	X	X
	Hrd3	−	−	−	−	X	−	X	X
	Uba1 (sUba1)	X	X	X	X	X	X	X	X
	Doa10	X	−	X	−	X	−	X	X
	Ubc6 (sUbc6)	X^sf^	−	X	−	X	X	X	X
	Polyubiquitin (sUbi)	X	−	X	−	X	X	−	−
Cdc48 complex	Cdc48 (sCdc48-1)	X	−	X	−	X	X	X	X
	(sCdc48-2)	−	−	−	−	−	X	−	−
	Ufd1 (sUfd1)	X	−	X	−	X	X	X	X
	Npl4 (sNpl4)	X	−	X	−	X	X	X	X
	(sUBX)	−	−	−	−	−	X	X	X
	(sPUB)	−	−	−	−	−	X	−	−
Processing	Png1 (sPng1)	X	X	X	X	X	X	X	X
	Dsk2 (sUbq)	X	X	−	X	X	X	X	X
	Rad23	X	−	X	−	X	−	X	X
	Ufd2	X	−	X	−	X	−	X	X
	(ptDUP)	−	−	−	−	−	X	−	−
Unknown function	(PPP1)	X	−	X	−	−	X	X	X
Chaperones	Hsp70 (sHsp70)	X	X^sp^	X	X	X	X	X	X
	Hsp40/Ydj1/MAS5	X	−	X	−	X	−	X	X
	(sDPC)	−	−	−	−	−	X	−	−

The genes of *N. oceanica* are searched against the genome obtained in this study, while those of *N. gaditana* are searched against the genome published early^19^ (e-value < e^−5^ and annotated as the genes searched). In parentheses, symbiont specific if with s or putatively symbiont specific if without s. X, found; –, not found; ?, not sure as the signal peptide at the N-terminal was not found except two with superscript sp of *N. oceanica*. sf, annotated as different names but the function is the same

^a^For these two species, host-specific (H) and symbiont-specific (S) are judged according to the similarity between protein query and its hit. Data cited from Stork et al.^42^

**Table 3:** The identification of translocon components in the outer and inner membranes (OM and IM) of plastid of *N. oceanica* (NO), *N. gaditana* (NG), *P. tricornutum* (PT), *C. merolae* (CM), and *G. sulphuraria* (GS)

Species		NO	NG	PT	CM	GS
Translocon in IM	Tic20	−	−	X	X	X
	Tic22	X	X	X	X	X
	Tic55	X	X	X	−	−
	Tic110	X	−	X	X	X
Translocon in OM	Toc75	−	−	−	X	X
	Omp85	X	X	X	X	X
	Toc64	X	X	X	X	X
	Toc34	X	X	X	X	X

The genes of *N. oceanica* are searched against the genome obtained in this study, while those of remaining species are searched against the genome published early. X, found; –, not found

### Nonmotility of *N. oceanica*

The flagellum is an axial 9 × 2+2 structural assembly that includes substructures such as dynein arms, radial spokes, and a central pair complex. Jang et al.^35^ described the existence of flagellum-associated genes in motile (mainly dinoflagellates), motile small (cryptophytes, euglenophytes, stramenopiles, and haptophytes), and nonmotile (stramenopiles, haptophytes, diatoms, and green algae) protists and found a nearly two-fold differential between motile and nonmotile protists (104 vs. 55). We found that the total numbers of flagellum-associated genes in *N. oceanica* and *N. gaditana* were 29 and 17, respectively (Table 4 and Supplementary Data 1), which explained to some extent the two most salient characteristics of *Nannochloropsis* species, their small size and nonmotility. The loss of flagellum-associated genes also reflected the reduction of *Nannochloropsis* genomes. From being motile to being small and motile to being completely nonmotile, the number of movement-associating genes is reduced exponentially. *Nannochloropsis* have lost their ability to move because their number of movement-associating genes is approximately one-fourth that of motile protists and approximately one-half that of small and nonmotile protists.

**Table 4:** The numbers of eight categories of flagellum-associated genes of two *Nannochloropsis* species and their comparison with those (in average) of protists determined early^35^

Gene category	Motile protists	Motile small protists	Nonmotile protists	*N. oceanica*	*N. gaditana*	Gene category
Structural gene	Tubulin	4	4	4	2	2
	Radial spoke	15	15	8	2	1
	Central pair	11	10	8	6	4
	ODA	19	19	11	6	4
	IDA	21	21	16	4	2
Functional gene	IFT-A complex	6	6	1	2	0
	IFT-B complex	21	20	6	7	4
	BBSome	8	8	2	0	0
Total	104	104	55	29	17	Total

Nonmotile species include stramenopiles, haptophytes, diatoms, and green algae; motile species are all dinoflagellates; motile small species include cryptophytes, euglenophytes, stramenopiles, and haptophytes. The genes of *N. oceanica* are searched against the genome obtained in this study, while those of *N. gaditana* are searched against the genome published early^19^ (searching with e-value < e^−5^ as the threshold of expected hits; filtrated with those of full lengths as candidates and accepted as the protein expected if a candidate shows the highest similarity which is usually extremely less than the e-value threshold for searching and is annotated simultaneously as the expected). ODA, outer dynein arm; IDA, inner dynein arm

### Loss of sexual reproduction

From mating types in yeast to different sexes in plants and animals and from a single gene to sex chromosome systems^36–39^ sex determination mechanisms have been intensively investigated. The sexual reproduction and characteristics of organisms can be extremely complex; however, mating, cell fusion, and meiosis are among the basic processes that are absolutely involved in sexual reproduction. We have tried to identify the mating and cell fusion-associating genes found in yeast^40^ and diatoms (phylogenetically closer to *Nannochloropsis*)^41^. Unfortunately, we found that this gene set is extremely diverse; thus, it is difficult to uncover the homologs of yeast and diatom cell mating and fusion genes among other organisms, including *N. oceanica* and *N. gaditana*. We turned to the second basic process involved in sex determination, the meiosis, focusing on the meiosis-specific genes developed as a sex detection toolkit^42,43^.

During evolution, the function of a gene may be replaced by other genes, and a gene may evolve a new function. We assumed that a meiosis-specific gene was truly absent and not replaced by an unidentified gene if it existed in a relatively wide taxonomic range of species. Of the nine meiosis-specific genes documented early^42,43^, *Rec8* was not identified in the two species of genus *Nannochloropsis*. A gene homologous to *Rec8* is found in their genomes; however, it is phylogenetically similar to *Rad21*, a mitosis-associating but not specific gene. The remaining eight meiosis-specific genes were identified with BLASTp or tBLASTn and by phylogenetic analysis in a yeast, two green algae, a brown alga and a haptophyte among the currently available genome sequences (Table 5; Supplementary Data 1). Although they do not completely encompass all species reproducing sexually, these species represent a relatively wide taxonomic range of species. Eight meiosis-specific genes are conserved among these species, indicating that they cannot be replaced and are not substitutable in meiosis. We failed to identify three meiosis-specific genes (*Hop1*, *Dmc1*, and *Mnd1*) in the genome of *N. oceanica* and two (*Hop1* and *Hop2*) in the genome of *N. gaditana* (Table 5). These findings (the absence of meiosis-specific and mating-associating genes) indicated that species in the genus *Nannochloropsis* have lost their ability to reproduce sexually.

**Table 5:** Identification of known meiosis-specific genes used as a meiosis detection toolkit

Species	Meiosis-specific genes^a^							
	*Spo11-2*	*Mer3*	*Hop1*	*Dmc1*	*Hop2*	*Mnd1*	*Msh4*	*Msh5*
*Saccharomyces cerevisiae*	X	X	X	X	X	X	X	X
*Chlamydomonas reinhardtii*	X	X	X	X	X	X	X	X
*Volvox carteri*	X	X	X	X	X	X	X	X
*Ectocarpus siliculosus*	X	X	X	X	X	X	X	X
*Emiliania huxleyi*	X	X	X	X	X	X	X	X
*Nannochloropsis oceanica*	X	X	−	−	X	−	X	X
*N. gaditana*	X	X	−	X	−	X	X	X

^a^Of nine meiosis-specific genes^51, 52^, *Rec8* is not identified in two species of genus *Nannochloropsis*. A homologous gene of *Rec8* is found in the genome of *N. oceanica*; however, it is phylogenetically similar to *Rad21*, a meiosis associating gene. We believed that a meiosis-specific gene is truly absent and not replaced by an unidentified gene if it exists in a wide taxonomical range of species which include here, for example, a yeast, two green algae, a brown alga, and a haptophyte. These genes are identified by searching against the genomes either downloaded from public databanks or sequenced ourselves (*N. oceanica*) with BLASTp or tBLASTn and by phylogenetic analysis

## Discussion

Heterokonta (or Stramenopiles) refers to the type of motile life cycle stage at which the flagellated cells possess two differentially shaped flagella. The typical classes in this infrakingdom^10^ include the colored Ochrophyta (alga-like) and colorless Pseudofungi and Bigyra phyla. The classes in the alga-like Ochrophyta include Dictyochophyceae (axodines), Bacillariophyceae (diatoms), Chrysophyceae (golden algae), Eustigmatophyceae, Phaeophyceae (brown algae), and Xanthophyceae (yellow-green algae), among others, while those in Pseudofungi include Oomycetes (water molds), among others^44^. However, the two most prominent characteristics, motile and flagellated cells, have never been observed among species of genus *Nannochloropsis*, class Eustigmatophyceae. This discrepancy between classification and observation prompted us to rethink the phylogenetic position of genus *Nannochloropsis* within phylum Ochrophyta.

We found that the phylogenetic positions of *Nannochloropsis* indicated by their 18S rDNA were different from those indicated by their whole genomes. The former implied an identical origin way to modern Chromista, while the latter implied an unusual way different from that of modern Chromista. If the nuclei of modern Chromista belong to diverse host protists, those of *Nannochloropsis* should belong to a protist different from all of them. We found that *Nannochloropsis* contained a similar number of red algal genes with diatoms and golden microalgae but obviously less genes of Haptophytes and Cryptophytes than diatoms and golden microalgae, indicating that a portion of *Nannochloropsis* genes should have been replaced by those from the symbiont nucleus. The positions of *N. oceanica*, *A. anophagefferens*, and *P. tricornutum* on phylogenetic trees are appropriate for determining the sources of genes; a gene of *N. oceanica* is from the host nucleus if it is phylogenetically near its homolog from *A. anophagefferens*, while a gene of *N. oceanica* is from the symbiont nucleus if it is phylogenetically far from its homolog in *A. anophagefferens*. We found that the nucleus of *N. oceanica* is more distant from that of *A. anophagefferens*. *N. oceanica* has more genes from the symbiont, thus its nucleus derived mainly from symbiont nucleus during speciation. As shown on rbcL phylogenetic and the whole-genome-based microevolutionary trees, the symbiont carries an ancient red algal chloroplast. Therefore, we believe that the symbiont is an ancient red alga that is phylogenetically distant from the current single-cell microalgae.

**Fig. 4:**
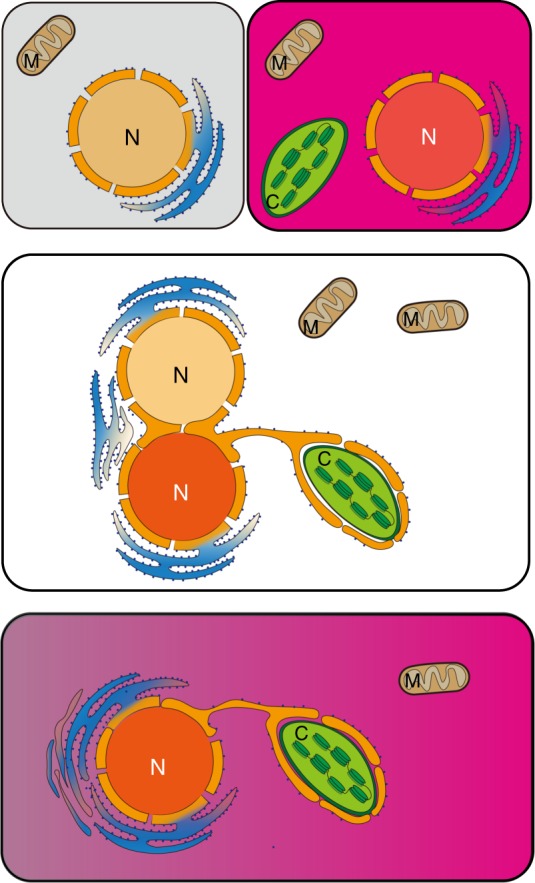
The hypothetical route of *N. oceanica* speciation through cellular fusion and nuclear haploidization. The symbiont nucleus may have overthrown the host nucleus during speciation of *Nannochloropsis*. The cell fusion mode of speciation needs no evolution of new structure and function, which should aid to fast speciation of *Nannochloropsis*. Instead of free floating, the plastid homing in the host cytoplasm may be enveloped by either one or two layers of cytoplasmic reticulum, forming a continuum with nucleus and dividing simultaneously with nucleus

The widely accepted understanding is that a protist engulfed and enslaved a red alga and discarded its nucleus but returned its chloroplast during secondary endosymbiosis, yielding cryptophytes, haptophytes, and heterokonts. During the speciation of *Nannochloropsis*, it seems that the host nucleus contributed only a small portion of the genes, including the rDNA, while the symbiont nucleus contributed a large portion of the genes. In other words, the symbiont nucleus has overthrown the host nucleus. We propose that *Nannochloropsis* evolutionarily emerged through the active fusion of a host protist and a symbiont photosynthesizing protist, forming a triad of two nuclei and a plastid; the two nuclei were covered by independent nuclear membranes and shared endoplasmic reticulum, while the plastid was covered by two layers of endoplasmic reticulum outside the two membranes derived from the primary endosymbiosis; and the two nuclei interacted with each other, the symbiont nucleus finally won the competition, and the host nucleus was overthrown during the speciation of *Nannochloropsis* (Fig. 4). The cell fusion mode of speciation requires no evolution of new structure and function, which should have helped *Nannochloropsis* to thrive during speciation.

We can find more clues among previous studies. For example, the nucleus and plastid of *N. oculata* accompany each other throughout the whole cell cycle by forming a nucleus-plastid continuum covered by a membrane with ribosomes on the outer surface^27^. This observation supports our hypothesis. The existence of barrel-shaped nuclear pole bodies^27^ in *N. oculata* evidence the ancient nature of the nuclei of *Nannochloropsis*, which may explain the unusual position of *Nannochloropsis* on the rbcL phylogenetic tree. In addition, it has been hypothesized previously that the two outermost membranes of complex plastids may both originate from the endoplasmic reticulum^45^.

Cell fusion and nuclear competition may avoid the formation of complex plastids and the requirement of new protein targeting systems. Complex plastids are believed to be veiled by four membranes originating during secondary endosymbiosis^45,46^. Early studies have demonstrated that translocons (or complexes) help to import proteins synthesized on the cytoplasmic ribosomes into the periplastid compartment and the stroma of the chloroplast^47–49^. From the outermost (possibly derived from the host endoplasmic reticulum) to the second outermost (periplastid membrane) and the inner plastid membranes, Sec61, SELMA, and TIC complexes have been suggested to import preproteins across these membranes; however, the TOC complex in the outer plastid membrane has not yet been found among complex plastids^50^. Instead of the TOC complex, a homolog of TOC75 (Omp85) has been demonstrated to mediate the translocation of proteins across the outer membranes of complex plastids^51^, and a Der complex has been proposed to replace TOC^52^. In addition, the genes encoding SELMA complex components are believed to be recycled from the red alga symbiont, and their corresponding proteins are synthesized in the cytoplasm and imported either into the periplastid compartment or the plastid stroma. These proteins are called preproteins and are marked by a bipartite topogenic signal at their N-terminals. A bipartite topogenic signal consists of a signal peptide for crossing Sec61, a transient peptide for crossing the periplastid membrane and a special amino acid residue between them for targeting the plastid stroma^50^. If the two outermost membranes of chloroplasts are derived from the endoplasmic reticulum, the evolution of complex preprotein transportation systems can be avoided. As was strongly expected, the plastids of *N. oceanica* and *N. gaditana* had not evolved a protein import system similar to that of *P. tricornutum*.

If *N. oceanica* and *N. gaditana* do not need these complexes, they must have adopted a membrane system different from that of *P. tricornutum* during evolution. Secondary endosymbiosis is always believed to be a passive process in which the host protist engulfs a photosynthesizing alga and enslaves its plastid but discards its mitochondria. This widely accepted mechanism hypothesized that: the newly appearing chloroplast must be covered by four membranes. The outermost membrane possibly originated from the endoplasmic reticulum, while the periplastid membrane possibly originated from the host cellular membrane, and the two interior membranes were inherited from the first endosymbiosis; a new system must import proteins synthesized in the cytoplasm into the periplastid compartment and the stroma of plastids; and the genes of the enslaved nucleus must undergo horizontal transfer to the host nucleus on a large scale. It seems that this mechanism has received strong support from the studies carried out in some Chromista^47–49^. However, this mechanism also has exceptions, including, for example, the absence of the components of the protein import system in many other species, including *N. oceanica* and *N. gaditana* as analyzed in this study. In our assumption of *N. oceanica* speciation, the chloroplast of *N. oceanica* was enveloped outside by two layers of endoplasmic reticulum, which is homogeneous to that found in the cytoplasm. Sec61 and ERAD should be involved in transportation into and out of the endoplasmic reticulum^45^. Although some modifications may exist, the protein transportation system of *Nannochloropsis* was inherited from the symbiont, e.g., an ancestral single-cell red alga. This evolutionary path would readily explain the speciation of *Nannochloropsis*.

The two nuclei fused to make a hybrid nucleus, which then reduced its genetic information by haploidization. During this kind of evolution, diverse structures and functions may have been lost through either selection or genetic drift or both, including motility and sexual reproduction. The flagellum plays important roles in competition for nutrients by enabling rapid movement, and thus, rapid finding, capture, and ingestion of prey. It also helps autotrophic and mixotrophic species sense and receive solar light. It is known that all species in the genus *Nannochloropsis* are extremely small, structurally simple and nonmotile^13^. The advantages and/or disadvantages of these characteristics in evolution remain unclear. However, these characteristics may imply the loss of structures, thus marking the reduction of the *Nannochloropsis* genome. In this study, we found that most flagellum-associating genes were lost, providing at least one example of such change during the speciation of *N. oceanica*.

The genome reduction of *N. oceanica* has also caused the loss of its other functions, including sexual reproduction. Neither ploidy variation nor morphological mating and cellular fusion has been documented among the species in the genus *Nannochloropsis* to date. As was expected, the function of sexual reproduction has been lost during the speciation of *N. oceanica*; an incomplete set of meiosis-specific genes were identified in the genome of *N. oceanica*. We once tried to determine the existence of sexual reproduction among microalgae with their genome sequences available by setting the number of meiosis-specific genes ≥ 6^53^. This early set threshold is roughly in accordance with what we found in this study. With the increase in available genome sequences, we believe that molecular characterization of the reproductive strategies of many organisms will become feasible and convenient.

## Methods

### Microalgal cultivation and DNA isolation

The marine microalga *N. oceanica* was provided by Key Laboratory of Mariculture of the Chinese Ministry of Education, Ocean University of China. The algae were cultivated in *f/2* medium (pH 7.8, salinity 30)^54^ prepared with natural seawater and autoclaved at 121 °C for 30 min. DNA was isolated with the cetyltrimethylammonium bromide (CTAB) method^55^.

### Genome sequencing

The whole genome of *N. oceanica* was sequenced on the PacBio Sequel System (https://www.pacb.com/products-and-services/pacbio-systems/sequel/) based on the single-molecule real-time (SMRT) sequencing technology. The template library was constructed using SMRTbell Template Prep Kit 1.0 (product code 100-259-100) and SMRTbell Damage Repair Kit (product code 100-465-900). Following the procedure described in the PacBio brochure to prepare > 20 kb template using BluePippin™ size-selection system (15–20 kb cutoff) for Sequel™ Systems, the quality DNA was fragmented with g-TUBE (covaries, 520079) and concentrated with AMPure^®^ PB magnetic beads with the fragments eluted with the Pacific Biosciences^®^ Elution Buffer. The fragments were damage-repaired with ExoVII, end-repaired with End Repair Mix and ligated with the blunt adapter. After removing away the failed ligation products with ExoIII and ExoVII, the ligation products were purified twice with AMPure^®^ PB Beads, and subjected to size selection using BluePippin™ Size-Selection System. The yielded fragments were bead-purified, damage-repaired, and used as the ~20 kb SMRTbell templates. The templates were annealed with primers and bound to DNA polymerase using the PacBio DNA/Polymerase Kit and magnetic beads, and loaded onto PacBio Sequel™ Systems to read the sequence of templates.

### Generation of short reads for genome correction

In order to collect the Illumina paired-end reads, the possible degradation and contamination of genomic DNA were observed in 1% agarose gel, the purity of the genomic DNA was determined on NanoPhotometer® (IMPLEN, CA, USA) and the concentration was measured with Qubit^®^ 2.0 Fluorometer (Life Technologies, CA, USA). The genomic DNA survived quality control was used to construct the short fragment library following the TruSeq DNA Sample Preparation Guide (Illumina, 15026486 Rev. C). The procedure includes mainly the steps of DNA fragmentation, end-repairing, base A tailing, adaptors ligation, recovering of DNA with desirable sizes from gel, and PCR amplification of the recovered DNA. The amplification products are used as the libraries for sequencing once they survived the quality checking. In brief, the amplification products were quantitated on Qubit2.0, and the size range of the amplification products was determined with Agilent 2100. If the fragment size ranged as the expected, the library was accurately quantified with Bio-RAD CFX 96 real-time quantitative PCR thermocycler and Bio-RAD KIT iQ SYBR GRN Q-PCR thermocycler. The quality library was sequenced on HiSeq X Ten Platform set at PE150 program with paired-end reads obtained.

### Genome assembling

The reads exported by Sequel™ Systems were quality evaluated with the in-built High Quality Region Finder (HQRF) which identifies the longest high-quality regain each read generated by a singly-loaded DNA polymerase according to the ratio of signal to noise. Upon being generated by the system, all bases were marked with exclamation point in order to make the format perfect. The quality reads (or regions) were marked with 0.8, while the low quality ones were marked with zero.

The quality reads obtained were assembled into contigs using Canu (v1.5 https://github.com/marbl/canu)^56^ by setting the parameters as the following: canu -pacbio-raw nanoc.subreads.bam.fasta -p nanoc -d nanoc -canu genomeSize=40m gridEngineMemoryOption=“-l vf=MEMORY”. Canu uses the all-versus-all overlap information to correct individual reads. It selects these overlaps in a two-step manor, global and local filtrations. The global filtration identifies the targets where a read may provide correction support, and the local filtration allows a read to accept or reject the correction evidences provided by other reads. At the trimming stage, Canu identifies the region of reads without correction supports, trimming or splicing the reads into their longest regions with correction supports. These regions were subjected to a final identification of sequencing errors, and used to construct the best overlap graph as the output contigs and make a summary statistics.

The errors in the primary assembly were identified and corrected with blasr (v5.1, https://github.com/PacificBiosciences/blasr)^57^ and Arrow (v2.2.1), a tool built in Smrt Link (https://downloads.pacbcloud.com/public/software/installers/smrtlink_5.0.1.9585.zip). First, the PacBio reads were mapped to the raw contig using blasr with the parameter: --bam --bestn 5 --minMatch 18 --nproc 4 --minSubreadLength 1000 --minAlnLength 500 --minPctSimila rity 70 --minPctAccuracy70 --hitPolicy randombest --randomSeed 1, and then the consensus sequences were obtained and variants were called via Arrow with the default parameters.

The consensus genome was subjected to a final round of base-error correction (polish) by referring to the Ilumina reads with BWA (v0.7.9a) and Pilon (v1.22, https://github.com/broadinstitute/pilon)^58^. The Illumina paired-end reads were mapped to the contigs by BWA (parameter, -k 30), and Pilon 1.22 (default parameters) uses this alignment to correct the assembly. The quality of the genome sequence obtained was further evaluated with BUSCO (v2, http://busco.ezlab.org/)^59^ (default parameters) based on a benchmark of 303 conserved eukaryota ortholog database.

### Construction of Hi-C library

Algal cells (0.2 g, fresh weight) were harvested through centrifugation and fixed with 2% formaldehyde to crosslink the DNA and proteins at a final concentration of 1% (v/v) and at room temperature for 15 min. The crosslinking was quenched with glycine at a final concentration of 0.2 M through incubation at room temperature for 5 min. The algal cells were collected and resuspended in cold 1× PBS. The cells were rinsed again and the cell pellet was flash-frozen in liquid nitrogen and stored at −80 °C.

The crosslinked algal cells were lysed in Hi-C lysis buffer (10 mM Tris-HCl pH 8.0, 10 mM NaCl, 0.2% Igepal CA630) supplemented with the protease inhibitors (Sigma, P8340) on ice for > 15 min. The debris (mainly nuclei) was collected through centrifugation (2500 × *g*, 5 min), washed with ice-cold Hi-C lysis buffer, gently resuspended in 0.5% sodium dodecyl sulfate (SDS) and incubate at 62 °C for 5–10 min, and then quenched with 10% Triton X-100 (Sigma, 93443) at 37 °C for 15 min. The chromatin was digested with *Mbo* I (New England Biolabs, R0147) overnight at 37 °C. The chromatin digestion was ceased by incubating at 62 °C for 20 min.

The cohesive ends of DNA were filled in and biotin labeled with DNA polymerase I Large (Klenow) Fragment (NEB, M0210) and biotin-14-dATP (Life Technologies, 19524-016) at 37 °C for 45 min. The DNA fragments were ligated proximately with T4 DNA ligase (NEB, B0202) at room temperature for 4 h. Crosslink was reversed by degrading the protein with proteinase K (NEB, P8102) when SDS was present at 55 °C for 30 min.

In order to extract HiC information through high-throughput sequencing using Illumina sequencers, the biotinylated DNA was sheared to sizes varying between 300 and 500 bp in length with Covaris LE220 (Covaris, Woburn, MA) (volume 130 μL in a Covaris microTUBE; fill level 10; duty cycle 15; PIP 500; cycles/burst 200; time 58 s). The sheared DNA was eluted and verified to be successfully sheared through agarose gel electrophoresis. The sheared DNA was purified with AMPure XP beads (Beckman Coulter, A63881). The DNA fragments in the range of 300–500 bp retained on the beads were eluted, quantified by Qubit dsDNA High Sensitivity Assay (Life Technologies, Q32854), and run on a 2% agarose gel to verify successful size selection.

The sheared DNA fragments were end-repaired with NEB T4 PNK (NEB, M0201), NEB T4 DNA polymerase I (NEB, M0203), and NEB DNA polymerase I Large (Klenow) Fragment (NEB, M0210) at room temperature for 30 min, and NEB Klenow exo minus (NEB, M0212) at 37 °C for 30 min. The DNA fragments containing biotin labels were pulled down and the biotin from unligated ends were removed away by adding to the reaction the pretreated Dynabeads MyOne Streptavidin T1 beads (Life Technologies, 65602). The Illumina indexed adapter was added to the DNA fragments attained on the beads, which were further polished with NEB Klenow exo minus (NEB, M0212) and ligated with an Illumina indexed adapter with NEB DNA Quick ligase (NEB, M2200) at room temperature for 15 min.

The HiC library was constructed by amplifying the innate one directly off of the T1 beads through PCR using Illumina primers and protocol, which was separated from the beads, purified with AMpure XP beads and eluted from the beads into 1× Tris buffer, yielding an in situ HiC library. The library was quantified and sequenced on an Illumina HiSeq 4000 Platform (San Diego, CA, USA) which reads 150 bp paired ends of DNA fragments.

### HiC information aided scaling-up of the genome assembly

A read was considered to be clean if it contained < 5 bases from adaptor; > 50% of bases with phred quality value of < 19; and < 5% of unknown base (N). The clean reads were first aligned on the genome assembly using the bowtie 2 (v2.2.3; http://bowtie-bio.sourceforge.net/bowtie2/index.shtml)^60^. The parameters used for bowtie were as the following: --very-sensitive -L 20 --score-min L,−0.6,−0.2 --end-to-end --reorder --rg-id BMG --phred33-quals -p 5. The unmapped reads were mainly composed of the chimeric regions spanning across the ligation junction. Following the philosophy of the Hi-C, the ligation site of an unmapped read was detected with HiC-Pro (v2.7.8; https://github.com/nservant/HiC-Pro)^61^ using an exact matching procedure and aligned its 5′ fractions back onto the genome. The results of both mapping steps were merged into a single alignment file, while the low mapping quality and multiple hitting reads and singletons were discarded.

The genome of *N. oceanica* was sequenced with the third-generation sequencing method. The contigs obtained cannot be scaled up to scaffolds; only one library was constructed. In this case, contig and scaffold are identical in meaning. The valid interaction pairs were used to build the interaction matrices and scale up the primary genome assembly in contigs to chromosome-scale scaffolds (hereafter pseudochromosomes) with LACHESIS^26^ (https://github.com/shendurelab/LACHESIS). The tool first clustered the contigs into chromosomal groups with the agglomerative hierarchical clustering algorithm and then ordered and oriented the contigs of each chromosomal group into pseudochromosomes. LACHESIS ordered the contigs by building a graph and extracting the longest path as the trunk (the highest-confidence order of the contigs in a chromosomal group). Those contigs excluded by the trunk were reinserted according to the linkage information between adjacent contigs. The ordered contigs were oriented by building a weighted, directed and acyclic graph (WDAG)^26^. The weight was calculated as the log-likelihood of Hi-C links between adjacent contigs^25^, and the orientation each contig each chromosomal group was predicted according to the maximum likelihood path through WDAG.

*N. gaditana* may contain 30 chromosomes, one chloroplast, and one mitochondrial chromosomes^62^. Accordingly, we set CLUSTER_N = 30 for LACHESIS. The tool then full-range scanned the five key parameters, including CLUSTER_MIN_RE_SITES [15,2000], CLUSTER_MAX_LINK_DENSITY [1,10], CLUSTER_NONINFORMATIVE_RATIO [1,v 10], ORDER_MIN_N_RES_IN_TRUNK [15,2000], and ORDER_MIN_N_RES_IN_SHREDS [15,2000]. All other parameters were set as the default. After about 2000 trials, a pseudochromosome candidate was picked up if it contained > 95% of the contig (in total length) and its corresponding trunk contained > 90% of the contigs (in total length). A single pseudochromosome was considered as the best if it contained 100% of the contig (in total length) and its corresponding trunk contained > 93% of the contigs (in total length). Finally, the tool yielded 29 nucleic pseudochromosomes, one chloroplast, and one mitochondrial pseudochromosomes.

To determine the accuracy of the scaling-up results, we cut the pseudochromosomes predicted by Lachesis into bins with 100 kb equal length and constructed the heatmap based on the interaction signals that were revealed by valid mapped read pairs between bins. The matrix was produced by Hic-Pro and then visualized as the heatmap to show the diagonal patches of strong linkage.

The plastid and mitochondrial genomes were clustered as two contig singletons, and both had long overlapping regions at both ends. This helped us to circularize their genomes. In addition, gene modeling and annotation characterized sets of genes shared by the chloroplasts and mitochondria of other species. To simplify chromosomal drawing, the chloroplast and mitochondrial genomes were linearized (Fig. 1a).

### Identification of repeat sequences

The tandem and interspersed repeats were recognized and annotated with RepeatProteinMask and RepeatMasker (v1.323, http://www.repeatmasker.org/) according to their sequence similarity with the deposited ones in Repbase^63^ (http://www.girinst.org/repbase/) and predicted as models with RepeatModeler (open-1.0.8, http://www.repeatmasker.org/RepeatModeler/) and de novo documented and annotated with RepeatMasker (v1.323, http://www.repeatmasker.org/). All the parameters were default. The tandem repeats were also predicted and annotated directly with TRF software^64^ built in RepeatMasker.

Repeats that were predicted and annotated in different ways were integrated and documented as nonredundant repeats. The tRNAs were predicted using tRNAscan-SE^65^ (http://lowelab.ucsc.edu/tRNAscan-SE/). Other noncoding RNAs, for example, rRNA, snRNA and miRNA, were identified by homologous searching for sequences deposited in the Rfam database (http://rfam.xfam.org/). All the parameters were default.

### Protein-encoding gene modeling and annotation

The genes in the genome assembly were modeled (predicted) following the philosophy described previously^66^. Such gene modeling procedure integrated the evidences from ab initio gene predictors based on Hidden Markov Models, spliced transcripts from RNA-seq data available currently and orthologous proteins from closely related species. Trinity (v2.4.0, default parameters)^67^ predicted and joined the potential transcripts. PASA (v2.1, https://github.com/PASApipeline/PASApipeline/wiki)^68^ extracted the open reading frames (ORFs) from these potential transcripts. These ORFs were directly used to optimize (train) the parameters of Augustus (v3.3, http://bioinf.uni-greifswald.de/augustus/), SNAP 2006-07-28 (http://archive.broadinstitute.org/mpg/snap/) and GlimmerHMM (v4.33, http://ccb.jhu.edu/software/glimmerhmm/) but not GeneMark-ES (v4.33, http://opal.biology.gatech.edu/GeneMark/) which used the genome assembly with the repetitive sequences marked to obtain an initial set of gene models^69,70^. Augustus was optimized for the parameters by eightfold cross-validation. SNAP and GlimmerHMM was optimized for the parameters by one round of training with the training set. GeneMark-ES did not require an explicit training data set. Orthologous protein sequences from *Nannochloropsis gaditana*^20^, *Phaeodactylum tricornutum*^71^, *Chlamydomonas reinhardtii*^72^, and *Chlorella variabili*^73^ from DOE-JGI (https://jgi.doe.gov) were mapped against the masked genome using tblastn 2.2.28 (https://blast.ncbi.nlm.nih.gov/Blast.cgi)^74^ (e-value = 1e−5), and then Genewise (v2.2.0, https://www.ebi.ac.uk/~birney/wise2/) was used to obtain the evidence of spliced protein-encoding gene. All the results were integrated into a single high-confidence gene model set with EVidenceModeler (v1.1.1, http://evidencemodeler.github.io/)^75^ using a weight set (PROTEIN GeneWise 5 ABINITIO_PREDICTION SNAP 1 ABINITIO_PREDICTION Augustus 5 ABINITIO_PREDICTION GlimmerHMM 1 ABINITIO_PREDICTION GeneMark.hmm 1 TRANSCRIPT assembler 10).

These genes were functionally annotated by homologous searching against Swiss-Prot (https://web.expasy.org/docs/swiss-prot_guideline.html), NCBI nt (https://www.ncbi.nlm.nih.gov/nucleotide/), NCBI nr (ftp://ftp.ncbi.nlm.nih.gov/blast/db/FASTA/nr.gz), Pfam (http://xfam.org/), eggnog (http://eggnogdb.embl.de/), GO (http://geneontology.org/page/go-database), and KEGG (http://www.genome.jp/kegg/).

### Phylogenetic analysis

The 18S ribosomal RNA (18S rDNA), ribulose bisphosphate carboxylase large chain (rbcL), meiosis-associated and flagellum component genes and genome sequences used in this study were retrieved from GenBank (https://www.ncbi.nlm.nih.gov/genbank/). A set of selected proteins or gene sequences was aligned with ClustalX (v2.0, http://www.clustal.org)^76^, and the phylogenetic tree of such set of sequences was constructed with MrBayes (v3.2.6, http://nbisweden.github.io/MrBayes/)^77^. The nuclear and chloroplast genomes selected for microevolution analysis included those of *C. merolae*^78^*, G. sulphuraia* ^79^, *C. reinhardtii*^72^*, C. variabilis* ^73^*, C. paradoxa*^80^*, E. huxleyi*^81^, *G. theta*^82^*, P. tricornutum*^71^*, A. anophagefferen*^83^, and *N. gaditana*^20^ from either NCBI (https://www.ncbi.nlm.nih.gov/) or DOE-JGI (https://jgi.doe.gov) and those of *N. oceanica* were obtained in this study. The OrthoFinder (v2.2.6, https://github.com/davidemms/OrthoFinder/releases)^84^ generated an orthogroup set showing the protein homologs among the species selected. The gene trees and species tree were inferred using the OrthoFinder workflow, including BLAST, MAFFT, and FastTree with green algae and Glaucophytes as outgroup. From the orthogroup set of all species, the orthogroups of *N. oceanica, P. tricornutum*, and *A. anophagefferens* were selected to clarify the similarities of protein homologs among these three species one by one. The protein homologs in orthogroups each were clustered using ClustalO (v1.2.3, http://www.clustal.org)^85^ by limiting the maximum number of sequences in sub-clusters to two. In this way, the most similar protein homologs between *N. oceanica* and diatom, between *N. oceanica* and golden alga and between diatom and golden alga can be identified and counted.

### Reporting summary

Further information on research design is available in the Nature Research Reporting Summary linked to this article.

## Supplementary information

Supplementary Information

Description of Additional Supplementary Files

Supplementary Data 1

Reporting Summary

## Data Availability

The *N. oceanica* genome assembly data are available through the NCBI under project number PRJNA503776. This genome assembly has been deposited at DDBJ/ENA/GenBank under accession number GenBank CP038106-CP03813.
