# Supplementary material for: Genome assembly of Nannochloropsis oceanica provides evidence of host nucleus overthrow by the symbiont nucleus during speciation
Source: Commun Biol. 2019 Jul 3;2:249. doi: 10.1038/s42003-019-0500-9 (PMC6610115; doi:10.1038/s42003-019-0500-9)
Supplement: Supplementary file 1 — Supplementary Information [file 42003_2019_500_MOESM1_ESM.pdf]

Supplementary Figures and Tables

Supplementary Figure 1

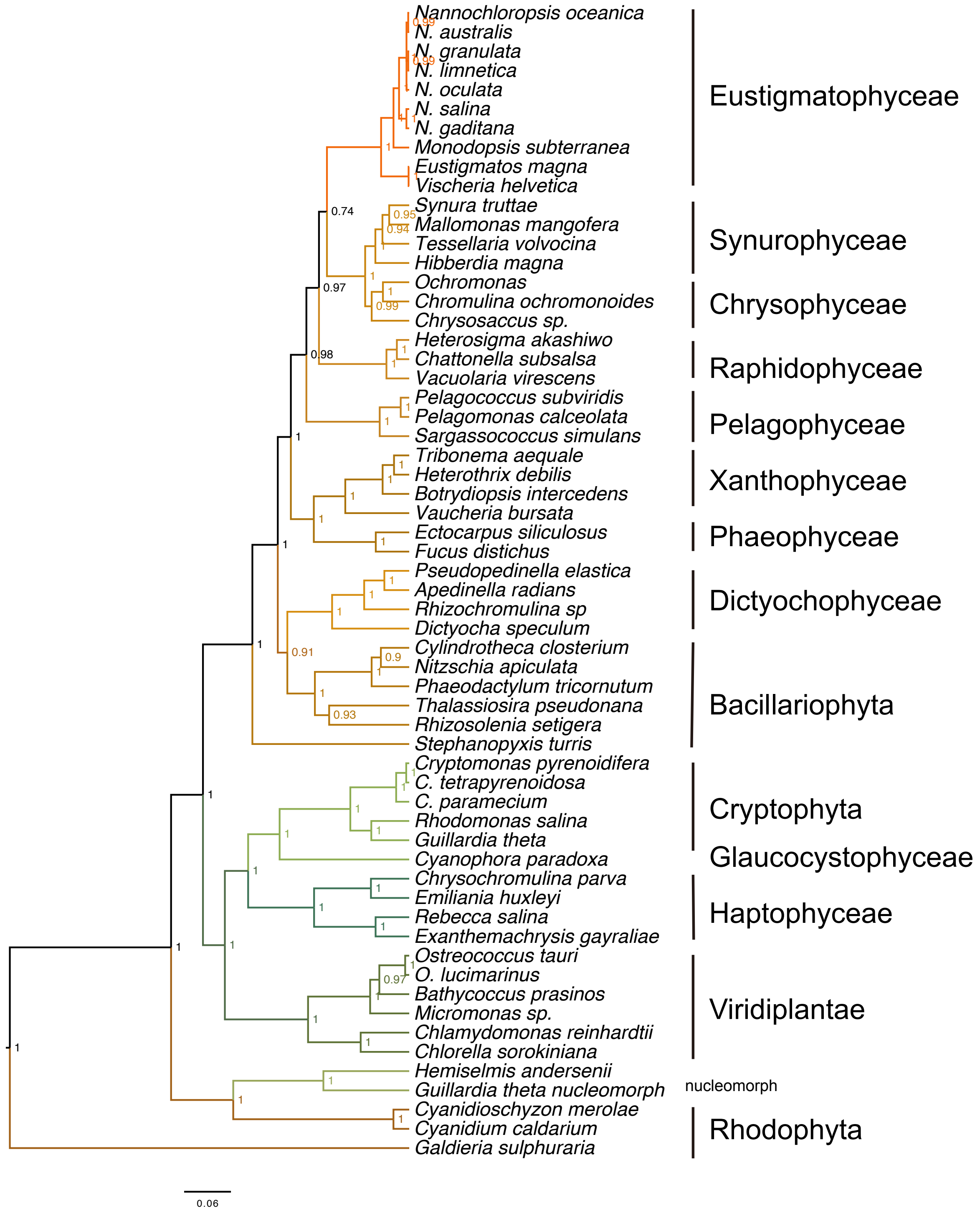

Supplementary Figure 1. The phylogenetic trees for18S rDNA. The tree shows the consensus tree topology inferred by Bayesian analysis using alignments of 18S genes from NCBI. The scale bar indicates the nucleotide substitutions per site. This consensus topology derived from 512 trees, lnL = 22033.73

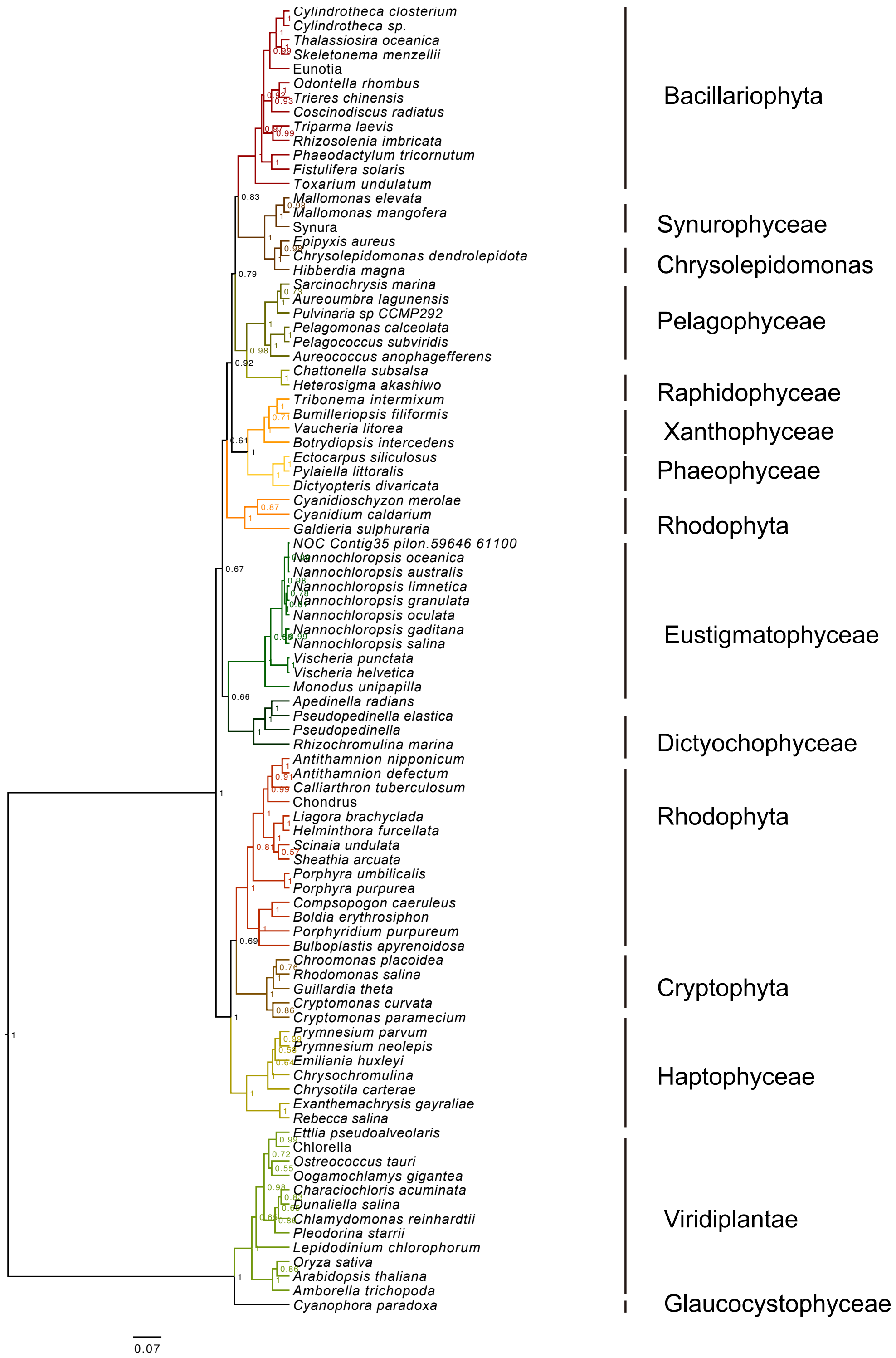

Supplementary Figure 2. The phylogenetic trees for rbcL protein. The tree shows the consensus tree topologies inferred by Bayesian analysis using alignments of rbcL proteins from NCBI. Scale bars represent 0.1 amino acid substitutions per site. In total, 439 aligned amino acid sites were analyzed. This consensus topology derived from 726 trees, α = 0.47 (0.41<α<0.56), pI = 0.0019 (0.0000007<pI<0.0059) and lnL = 10364.8.

Supplementary Table 1 Statistics of *Nannochloropsis oceanica* genome assembly

Contig	number
Assembled genome size (bp)	29,303,273
Read coverage depth (fold)	112
No. of contigs	129
Contig N_{50} (bp)	664,749

Supplementary Table 2 Clustering of contigs (scaffolds*)

Statistics	Result
No. of contigs	129
Length of genome (bp)	29,303,273
No. of contigs clustered	129
Rate of contigs clustered (%)	100
Length of contigs clustered (bp)	29,303,273
Rate of genome clustered (%)	100

*, the genome of *N. oceanica* is sequenced with so-called the third generation sequencing techniques, which do not read the DNA molecules with different lengths, and the genome assembling yields the continuous DNA sequences corresponding to the contigs of the second generation sequencing techniques. These contigs are not upgraded into scaffolds, thus they can be called either contigs or scaffolds, here contigs for the convenience of description.

Supplementary Table 3 Orientation and ordering of contigs*

Statistics	Result
No. of contigs ordered	128**
Rate of contigs ordered (%)	99.22
Length of contigs ordered	28,129,022
Rate of contig length ordered (%)	95.99
No. of contigs in trunks	86***
Rate of contigs in trunks (%)	67.19
Length of contig length in trunks	26,395,858
Rate of contig length in trunks (%)	93.84

*, the word contig is used directly.
**, one contig is a singleton which is not related with any other contigs.
***, a trunk contains at least more than three contigs.

Supplementary Table 4 The final assembly of *N. oceanica* genome with the assistance of HiC sequencing

	Length of contigs (bp)	Length of pseudo-chromosomes (bp)	No. of contigs	No. of pseudo-chromosomes*
Total	29,303,273	29,312,973	129	32
Max. length	1,540,838	1,670,642	-	-
No. contigs or pseudo-chromosomes ≥ 2, 000 bp	-	-	129	32
N50	664,749	1,148,430	15	11
N60	422,361	961,379	20	14
N70	350,531	877,916	27	17
N80	195,573	814,094	38	20
N90	82,245	585,400	62	25

*, the nucleus contains 30 pseudo-chromosomes, and mitochondrion and chloroplast contain one pseudo-chromosome each.

Supplementary Table 5 Number of protein coding genes predicted with different strategies and their characteristics

		No. of genes	Length (bp)	CDS (bp)	No. of exons	Length of exons (bp)	Length of introns (bp)
Strategy 1	Augustus	6594	2456.0	554.6	2.75	554.6	533.9
	GlimmerHMM	751	34043.0	455.7	12.58	455.7	2447.6
	GeneMark	6620	2893.4	273.9	4.94	273.9	391.5
	SNAP	9143	1128.1	347.1	2.3	347.1	253.9
Strategy 2	*N. gaditana*	7708	1075.3	320.2	2.47	320.2	195.1
	P. tricornutum	4663	848.7	336.4	1.99	336.4	184.2
	C. reinhardtii	3999	800.1	339.5	1.86	339.5	194.4
	C. variabilis	4064	799.0	340.8	1.85	340.8	197.0
	Available transcripts	7905	2002.6	626.7	1.88	887.1	374.8
Integrated	EVidenceModeler	7330	2084.0	483.7	2.87	483.7	370.9

The gene prediction was carried out with two strategies. With strategy one, the genes were predicted according to their characteristics (modeling) with diverse tools. With strategy two, the genes were predicted based on their similarities with the homologs in different known genomes found by searching with tools like blast and Genewise. All the predicted genes were integrated and documented as the non-redundant with EVidenceModeler. The predicted genes were characterized in their average length (bp), average CDS length (bp), average lengths of exon and introns (bp) and average numbers of exons.

Supplementary Table 6 Statistics of non-protein coding RNA

Type		Copy no.	Average length (bp)	Total length (bp)	% of genome
miRNA		15	129.9	1948	0.00665
tRNA		105	81.2	8525	0.02909
rRNA	18S	16	380.3	6084	0.02076
	28S	8	101.5	812	0.00277
	5.8S	4	155.0	620	0.00212
	5S	9	119.0	1071	0.00365
snRNA	CD-box	3	146	438	0.00149
	HACA-box	0	0	0	0
	Splicing	2	129	258	0.00088

Supplementary Table 7 Statistics of repeated sequences predicted with different tools

Prediction tool	Total length of repeats (bp)	Percentage in genome
RepeatMasker	3,306,419	11.28
ProteinMask	4,202,351	14.34
de novo	4,438,123	15.15
Trf	1,536,969	5.25
Total	5,719,313	19.52

Of the repeated sequences, tandem and interspersed repeats occupied 5% and 7.71% of the genome, respectively. Of the interspersed repeats, transposons occupied 4% of the genome while the long interspersed and long terminal repeats occupied 1.62% and 2.09% of the genome, respectively. Two types of transposons, CMC-EnSpm and MULE-MuDR were predominant; they had 5142 and 1049 copies, respectively. More repeated sequences were recognized in the genome of *N. oceanica* than in that of *N. oceanica* CCMP1779, which may be due to longer contigs obtained in this study than those obtained early (Vieler *et al.*, 2012).

Supplementary Table 8 Length (bp) and percentage of classified repeated sequences in *N. oceanica* genome

	Repbase TEs		TE proteins		RepeatModeler		Combined TEs	
	Length	Percentage	Length	Percentage	Length	Percentage	Length	Percentage
DNA	0	0	134,499	0.46	1,037,904	3.54	1,171,167	4.00
LINE	0	0	208,839	0.71	265,698	0.91	474,305	1.62
SINE	0	0	0	0	0	0	0	0.00
LTR	0	0	574,434	1.96	39,156	0.13	613,154	2.09
Other	0	0	117	0	585,003	2	585,120	2.00
Unclassified	3,306,419	11.28	3,313,337	11.31	2,596,495	8.86	3,452,335	11.78
Total	3,306,419	11.28	4,202,351	14.34	4,438,123	15.15	5,446,334	18.59

LINE, long interspersed nuclear elements; SINE, short interspersed nuclear elements; LTR, long terminal repeats.

Supplementary Table 9 Functional annotation of *N. oceanica* gene models against different data bases

Database	No. of models	Percentage
NT	477	6.51
GO	4776	65.16
Map	1969	26.86
BLASTP	4835	65.96
NR	6814	92.96
PFAM	5194	70.86
Eggnog	3783	51.61
BLASTX	4663	63.62
KO	3130	42.70
Total (non-redundant)	7330*	--

*, a portion of genes are annotated as either putative or theoretical proteins.
