# Supplementary material for: Genome assembly of Nannochloropsis oceanica provides evidence of host nucleus overthrow by the symbiont nucleus during speciation
Source: Commun Biol. 2019 Jul 3;2:249. doi: 10.1038/s42003-019-0500-9 (PMC6610115; doi:10.1038/s42003-019-0500-9)
Supplement: Supplementary file 2 — Description of Additional Supplementary Files [file 42003_2019_500_MOESM2_ESM.docx]

**Description of Additional Supplementary Files**

**File name**: Supplementary Data 1

**Description**: The source data underlying the graphs and charts were presented in Table 2,3,4,5 and Fig. 3.

The numbers of eight categories of flagellum-associated genes of two Nannochloropsis species were presented in Table 2. The identification of translocon components in IM and OM of plastid were presented in Table 3. The identification of host ERAD and symbiont SELMA components in *N. oceanica, N. gaditana, P. tricornutum, C. merolae* and *G. sulphuraria* were presented in Table 4. Identification of known meiosis-specific genes used as a meiosis detection toolkit were presented in Table 5. The percentages of homologous genes *P. tricornutum, T. pseudonana, A. anophagefferens, N. oceanica* and *N. gaditana* found in the nuclear genomes of *E. huxleyi, G. theta, C. merolae* and *G. sulphuraria* were presented in chart named Fig.3.
